# Valorization of Natural Cardio Trekking Trails Through Open Innovation for the Promotion of Sustainable Cross-generational Health-Oriented Tourism in the Connect2Move Project: Protocol for a Cross-sectional Study

**DOI:** 10.2196/39038

**Published:** 2022-07-13

**Authors:** Barbara Mayr, Maximilian Beck, Laura Eisenberger, Verena Venek, Christina Kranzinger, Andrea Menzl, Bernhard Reich, Veronika Hornung-Prähauser, Renate Oberhoffer-Fritz, Birgit Böhm, Josef Niebauer

**Affiliations:** 1 Institute of Sports Medicine, Prevention and Rehabilitation and Research Institute of Molecular Sports Medicine and Rehabilitation Paracelsus Medical University Salzburg Austria; 2 Ludwig Boltzmann Institute for Digital Health and Prevention Salzburg Austria; 3 Institute of Preventive Pediatrics Faculty of Sport and Health Sciences Technical University of Munich Munich Germany; 4 Salzburg Research Forschungsgesellschaft mbH Salzburg Austria; 5 St. Irmingard Klinik Prien Clinic for Cardiology Prien am Chiemsee Germany

**Keywords:** cardiorespiratory fitness, exercise, field test, hiking safety

## Abstract

**Background:**

Hiking is one of the most popular forms of exercise in the alpine region. However, besides its health benefits, hiking is the alpine activity with the highest incidence of cardiac events. Most incidents occur due to overexertion or underestimation of the physiological strain of hiking.

**Objective:**

This project will establish a standardized cardio trekking test trail to evaluate the exercise capacity of tourists within hiking areas and deliver a tool for the prevention of hiking-associated cardiac incidents. Further, individual exercise intensity for a hiking tour will be predicted and visualized in digital maps.

**Methods:**

This cooperation study between Austria and Germany will first validate a 1-km outdoor cardio trekking test trail at 2 different study sites. Then, exercise intensity measures on 8-km hiking trails will be evaluated during hiking to estimate overall hiking intensity. A total of 144 healthy adults (aged >45 years) will perform a treadmill test in the laboratory and a 1-km hiking test outdoors. They will wear a portable spirometry device that measures gas exchange, as well as heart rate, walking speed, ventilation, GPS location, and altitude throughout the tests. Estimation models for exercise capacity based on measured parameters will be calculated.

**Results:**

The project “Connect2Move” was funded in December 2019 by the European Regional Development Fund (INTERREG V-A Programme Austria-Bavaria – 2014-2020; Project Number AB296). “Connect2Move” started in January 2020 and runs until the end of June 2022. By the end of April 2022, 162 participants were tested in the laboratory, and of these, 144 were tested outdoors. The data analysis will be completed by the end of June 2022, and results are expected to be published by the end of 2022.

**Conclusions:**

Individual prediction of exercise capacity in healthy individuals with interest in hiking aims at the prevention of hiking-associated cardiovascular events caused by overexertion. Integration of a mathematical equation into existing hiking apps will allow individual hiking route recommendations derived from individual performance on a standardized cardio trekking test trail.

**Trial Registration:**

ClinicalTrails.gov NCT05226806; https://clinicaltrials.gov/ct2/show/NCT05226806

**International Registered Report Identifier (IRRID):**

DERR1-10.2196/39038

## Introduction

Regular physical activity improves exercise capacity [1,2] and reduces the risk of developing cardiovascular diseases [3]. Endurance and strength training have the highest levels of evidence in current national and international guidelines as therapeutic components for the prevention and rehabilitation of cardiovascular diseases [4]. Hiking is a popular mode of exercise in mountainous regions worldwide and is being performed by millions of people, including those with an increased cardiovascular risk [5]. Despite its well-known beneficial effects on the cardiovascular system, intensive and exhaustive physical activities can lead to cardiac events, especially in untrained individuals. As a result, hiking is the alpine activity with the highest incidence of cardiac events, including sudden cardiac death [6-8]. These critical incidents occur mostly due to overestimating personal fitness and choosing overly demanding hiking routes or neglecting weather conditions (temperature, wind, rainfall, snow, etc). In few cases, hikers know about their higher risk and prevent incidents during hikes through medical prevention check-ups beforehand. Such a check-up for heart health and an evaluation of the exercise capacity during standard conditions could ideally be done by a simple ergometry test. Based on its results, training and hiking recommendations could be given by an expert. To further enhance the safety of hiking as a means of preventing cardiovascular disease and helping prevent cardiac incidents, this study aims to develop and validate a standardized 1-km cardio trekking test trail (CTTT) that can be set up in mountainous areas to determine individual physical fitness levels and personalize categories of exercise intensities during walking and hiking. Additionally, we will perform scientific mapping of 2 cardio trekking trails based on several performance parameters, such as heart rate and gas exchange. The realization of those study aims should not substitute the medical prevention examination but rather provide an additional prevention method to predict cardiovascular demand in individual hikers.

This study is part of a larger project that aims to appreciate natural and evidence-based cardio trekking trails through open innovation methods for the sustainable promotion of cross-generational health-oriented tourism, called “Connect2Move.” The project has been funded by the European Regional Development Fund (INTERREG V-A Programme Austria-Bavaria – 2014-2020; Project Number AB296). This project aims to redesign existing hiking routes into themed trails and digitally redesign those trails. Expected cardiovascular load based on individual cardiorespiratory fitness will be visualized in digital maps, in addition to the usual description of the length, altitude, path condition, and duration. For valorization and implementation in the participating communities, an open innovation approach is chosen, which involves regional stakeholders and the target population early in the development process, and is scientifically and medically supervised. Two cross-border, climate-friendly, biological concepts for physical activity promotion/cardio trekking in the Alps will be developed. The ideas can increase year-round gentle health tourism and promote individual health literacy for tourists and locals.

## Methods

### Study Design

**Figure 1:**
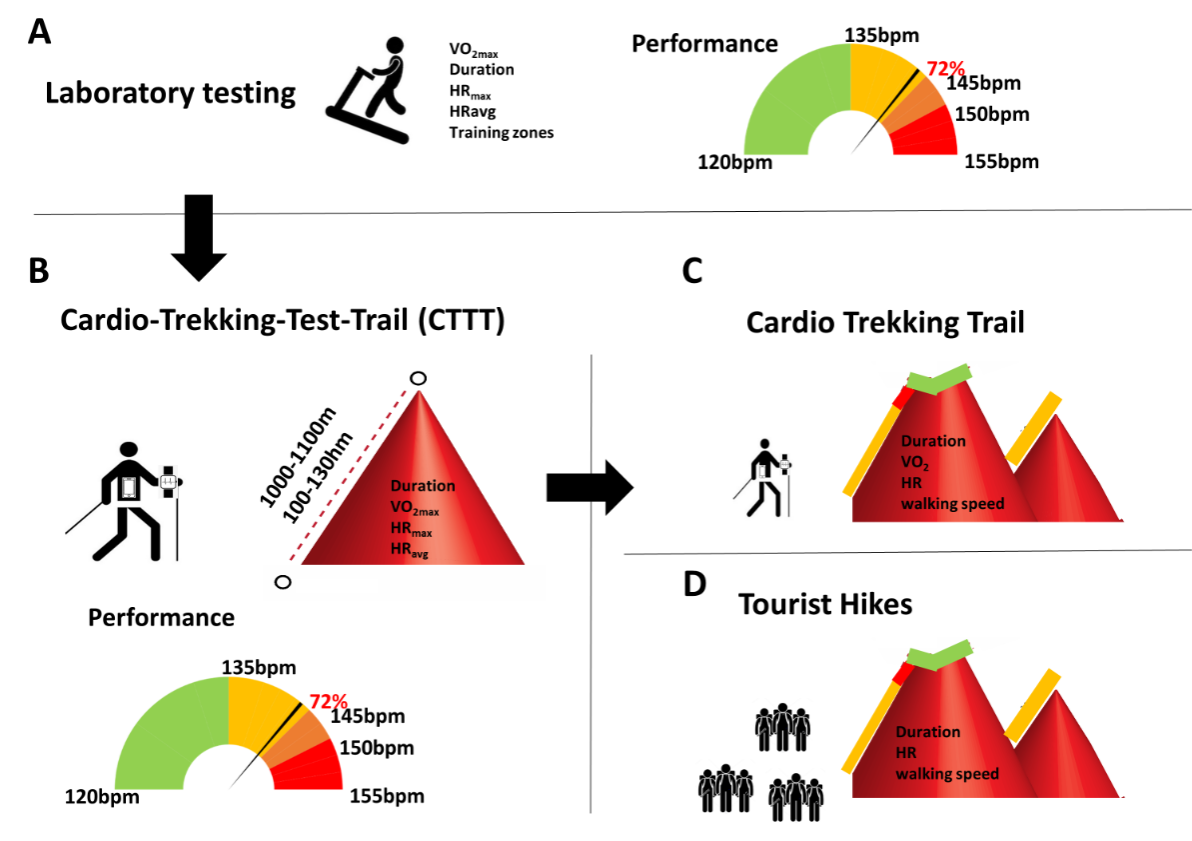
From laboratory testing to tourist hikes. (A) Laboratory treadmill test. Evaluation of maximal exercise capacity and training zones. (B) 1-km cardio trekking test trail. Evaluation of exercise capacity in a standardized outdoor setting. (C) 8-km cardio trekking trail. Guided hikes with study participants, with measurement of exercise capacity and heart rate during the hike. (D) Cartography of the 8-km cardio trekking trail with the help of guided tourist hikes. bpm: beats per minute; HR: heart rate; HR_avg_: average heart rate; HR_max_: maximal heart rate; VO_2_: oxygen uptake; VO_2max_: maximal oxygen uptake.

This cross-sectional study to reduce the risk of cardiac events in hiking consists of 3 work packages. It will be conducted in Salzburg and Werfenweng, Austria, as well as in Prien am Chiemsee and Aschau im Chiemgau, Germany. The first work package establishes and validates a 1-km CTTT. The second work package analyzes the physiological aspects of an 8-km trekking trail (Figure 1). In the third work package, developing an algorithm to calculate exercise intensity based on the results of the CTTT is the main task. This work package includes the cartography of intensities over the 8-km trekking trail. The first and second work packages will be performed by the Ludwig Boltzmann Institute for Digital Health and Prevention Salzburg and the Technical University Munich, and the third work package will be performed by Salzburg Research. The laboratory testing and medical assessment of the Austrian participants will be performed at the University Institute of Sports Medicine, Prevention and Rehabilitation, Paracelsus Medical University Salzburg, Austria, and the testing and assessment of the German participants will be performed at St. Irmingard Klinik in Prien am Chiemsee, Germany.

### Ethics Approval

Inclusion and exclusion criteria are described in Textbox 1. Participants will have to provide written informed consent before participating in the study. The Ethics Committee of the State of Salzburg (EK-Nr.: 1090/2020) and the Ethics Committee of the Medical Faculty of the Technical University of Munich (527/20S) have approved this study. Further, the study has been registered at ClinicalTrials.gov (NCT05226806). The study will be conducted following the ethical guidelines of the Declaration of Helsinki.

**Inclusion and exclusion criteria.**

**Inclusion criteria**

- Age >45 years
- Any sex
- Signed informed consent
- No relevant pathologies found during initial laboratory testing

**Exclusion criteria**

- Acute or chronic cardiovascular diseases, including untreated or insufficiently treated arterial hypertension (systolic blood pressure ≥140 mmHg or diastolic blood pressure ≥90 mmHg)
- Acute or chronic lung diseases
- Liver diseases
- Kidney diseases
- Diabetes mellitus
- Alcohol (>30 g/day) or drug abuse
- BMI >35
- Orthopedic restriction precluding physical activities performed during the study
- Pregnancy

### Recruitment Process and Measurement Process

The recruitment of participants will be conducted using the different information channels of the included institutes via the project website, recruitment flyers, and word-of-mouth advertising. If potential participants are >45 years of age, they will be informed about the study objectives, evaluation protocol, and procedures. On agreeing to participate, they will be invited to the initial laboratory testing at 1 of the 2 testing sites. At the beginning of the initial testing, researchers will provide detailed information about the study again, and the participants will be asked to read and sign written informed consent.

Participants will have to perform 3 different exercise testing procedures to complete the study, including 1 laboratory test on a treadmill and 2 field tests (1-km CTTT and 8-km hikes) in Werfenweng, Austria, or Aschau im Chiemgau, Germany. There will be at least 1 full day of rest between the laboratory test and the first field test. The 1-km CTTT and 8-km hikes will be performed on 2 days. If the scheduling of 2 days is not possible, the 1-km CTTT and 8-km hikes will be conducted on the same day with at least 1 hour of rest in between. Half of the study population will participate in Austria, and the other half will participate in Germany.

### Laboratory Testing

The baseline testing will start with a detailed examination by a specialist in internal medicine. Measurements will include anthropometrics (weight, height, and BMI), pulmonary function testing (spirometry), resting electrocardiography (ECG), blood pressure, patient history, echocardiography, and blood testing (lipids, electrolytes, markers of kidney and liver function, thyroid hormones, glucose, and blood count), as well as risk scores for cardiac events in the next 10 years, including the prospective cardiovascular Münster study (PROCAM) Score [9], Framingham Score [10], and European Society of Cardiology (ESC) Score [11]. Other health information will be collected via a face-to-face interview by trained staff. It will include personal questions regarding smoking and alcohol consumption, and sociodemographic data (education, employment status, and marital status). Participants will be asked to fill out standardized questionnaires on physical activity (International Physical Activity Questionnaire [IPAQ] short form [12]) and health (Health Survey Questionnaire: 36-Item Short Form Health Survey [SF-36] [13]). Before exercise capacity is measured, it will be estimated based on a resting test protocol called the Polar Fitness Test, using the Polar beat app and a chest-strap ECG-based heart rate monitor (Polar Electro). The last part of the baseline testing will be an all-out spiroergometry test on a treadmill. The participants will wear the portable spirometry device K5 (Cosmed) to measure respiratory gas exchange throughout the test. The gas analysis will occur in the K5’s dynamic mixing chamber (DMC) mode. Heart rate will be measured using a Garmin chest strap. In addition, the participants will wear a 12-lead ECG device (Amedtec Medizintechnik Aue GmbH) for evaluation of exercise-dependent ECG changes, as well as a Garmin Vivoactive 4 smartwatch, which will measure wrist-based heart rate values from an optical sensor throughout the treadmill test. All 3 measurement methods for heart rate will be started simultaneously. The modified Bruce protocol [14] will be used as the treadmill test protocol and will start with 3 min at 2.4 km/h and 0% incline, followed by 3 min at 2.4 km/h and 5% incline. After the first 2 stages, the traditional Bruce protocol will be followed. The test will be stopped if participants reach maximal exhaustion or start running. At the end of each stage, the participants will be asked to indicate their rate of perceived exertion (RPE) on a printed 6-20 Borg scale [15], including a maximum value at test termination. Based on the spiroergometry results measured via the K5 device, ventilatory thresholds for each participant will be determined by the V-slope method [16]. Exercise recommendations (heart rate zones) for the 8-km hike will be made based on these thresholds.

### Environmental Factors During Field Tests

To keep the temperature difference between the field tests and the spiroergometry in the laboratory as little as possible, the laboratory test will be carried out at a standardized room temperature of 20°C, which corresponds with the expected average daily temperature of the study months in the test regions. Should temperatures exceed 26°C, the tests will be postponed since a relevant impact on the heart rate response would be expected [17]. Moreover, humidity will be recorded.

### CTTT (1 km)

**Figure 2:**
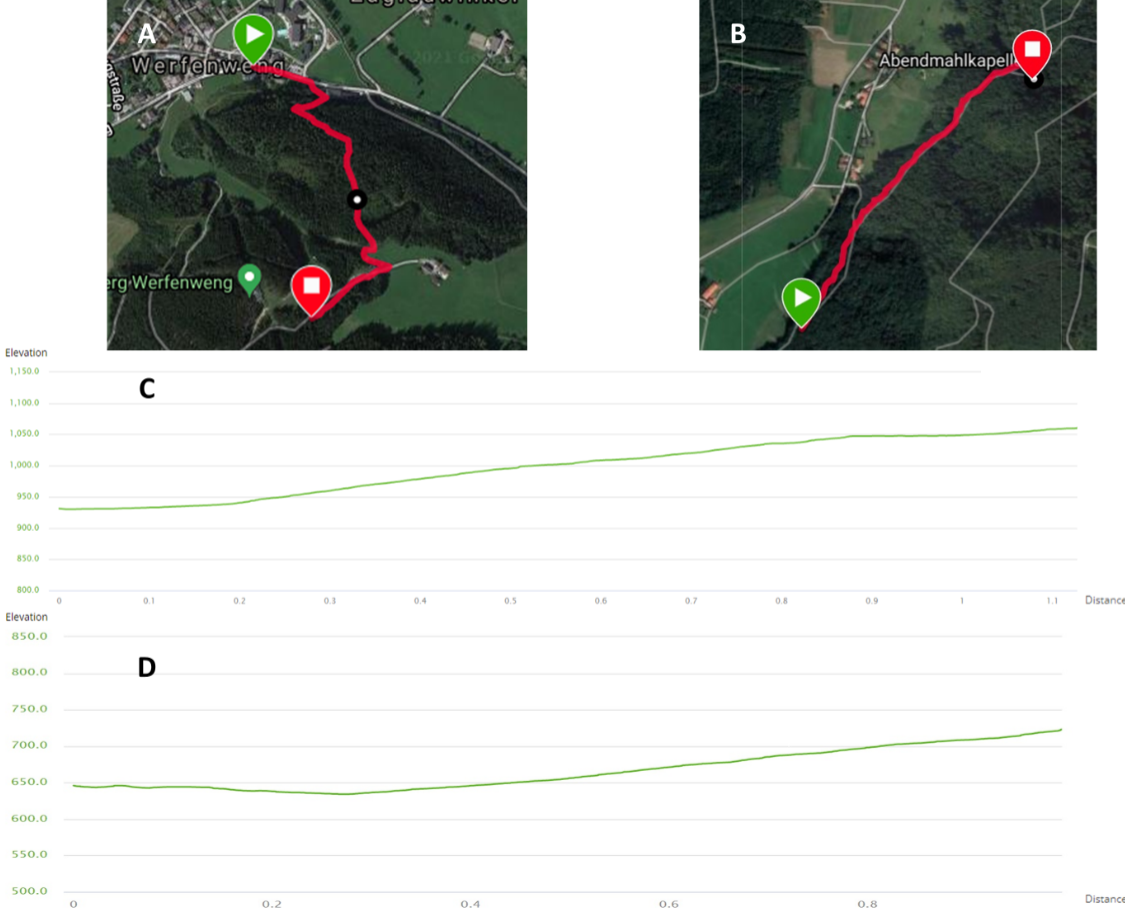
Cardio trekking test trail profiles. (A) Map of the cardio trekking test trail in Werfenweng, Austria. (B) Map of the cardio trekking test trail in Aschau im Chiemgau, Germany. (C) Profile of the Austrian trail. (D) Profile of the German trail.

The CTTT will be implemented outdoors in Werfenweng, Austria, and Aschau im Chiemgau, Germany. The maps and height profiles of the chosen trails for the CTTT are shown in Figure 2. The CTTT includes an easily accessible path with a length of 1000 (SD 100) meters and an elevation of 100 (SD 30) vertical meters, as well as a gradient of up to 26%.

After completing the laboratory test, all participants who have passed the baseline examination (no relevant heart diseases found during testing) will be invited for the field testing. This submaximal cardiorespiratory fitness test will be performed on the 1-km CTTT by each participant alone, with a researcher walking beside the participant. After an explanation of the route, as well as how to walk the track, the participants will get equipped with the measuring devices using the same setup as that during the treadmill test, except for the 12-lead ECG device. They will be wearing the portable K5 device operating in DMC mode, the Garmin chest strap, which will send their heart rate to the K5 device, and the Garmin Vivoactive 4 smartwatch set for wrist-based heart rate measurement. The outdoor exercise test should not be an all-out assessment but rather a submaximal assessment. A researcher will individually accompany participants (walk by their side) and guide their intensity via the Borg scale. Participants will be instructed to hike at a brisk pace and adjust their walking speed according to the trail’s steepness. They will be further required not to reach >15 RPE on the Borg scale. Individual Borg values will be assessed at multiple stages of the CTTT, and the pace will be adjusted accordingly if the RPE value exceeds 15 or falls below 11.

### Field Test Involving 8-km Hikes

**Figure 3:**
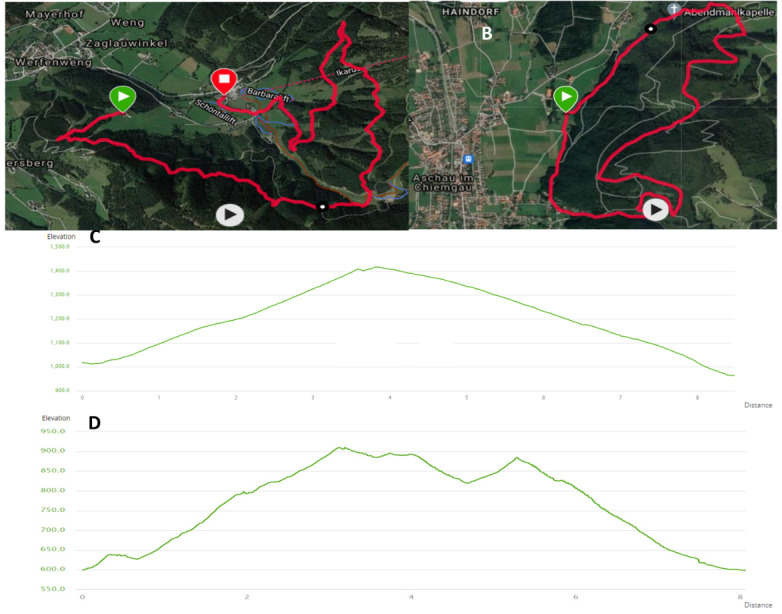
Cardio trekking trail profiles. (A) Map of the cardio trekking trail in Werfenweng, Austria. (B) Map of the cardio trekking trail in Aschau im Chiemgau, Germany. (C) Profile of the Austrian trail. (D) Profile of the German trail.

The 8-km hikes were selected based on touristic, sport scientific, and medical aspects like accessibility, visitor frequency, difficulty, soil texture, important landmarks for sightseeing, etc. Moreover, proximity to the selected CTTT plays an essential role in choosing the tracks. The maps and height profiles of the chosen trails for the long hikes are shown in Figure 3. The selected course in Austria has a length of approximately 8400 m, with 402 vertical meters uphill and 461 vertical meters downhill, and the steepest passage has a 37% gradient. The German trail has a length of approximately 8200 m, with 415 vertical meters uphill and 410 vertical meters downhill, and the steepest passage has a 26% gradient.

For the clinical study, we plan for at least one-third of all participants to hike the 8-km trail wearing the portable K5 device in the DMC mode. The Garmin chest strap sends heart rate data to the K5 device, and the Garmin Vivoactive 4 smartwatch is set for wrist-based heart rate measurement. All participants, who will hike the long trail without the K5 device, will track their hike using a Garmin chest strap connected to the Garmin Vivoactive 4 smartwatch. The groups for the long hike will vary in group size from 2 (1 researcher and 1 participant) to 6 (1 researcher and up to 5 participants). Intensity on the 8-km hike will be moderate. The participants will get individual heart rate ranges based on their ventilatory thresholds assessed during the laboratory treadmill test. Throughout the hike, the heart rate should remain in that range. In addition, the researcher will determine RPE occasionally, which should not exceed a value of 15 on the Borg scale.

### Cartography of the 8-km Cardio Trekking Trails

Subsequent to the 8-km hikes with the study participants, we will perform additional “cardio hikes” for tourists to gain further data for georeferencing and cartography of the selected hikes. We aim to complete guided hikes for 250 willing participants in both countries. All participants will be outfitted with heart rate sensors (Polar Verity Sense arm strap or Garmin Vivoactive 4 smartwatch) for tracking their heart rate, speed, etc, during the hikes. The selected 8-km cardio hikes in Austria and Germany will be newly mapped based on the data from our study participants. This part of the project will aim to tone the hike sections according to heart rate.

### Primary and Secondary Outcomes

#### Primary Outcome

The primary outcome is estimating the exercise capacity based on physiological features, subjective performance parameters, and route characteristics collected for 2 cardio trekking test routes in Werfenweng, Austria, and Aschau im Chiemgau, Germany. The 2 test routes have a length of approximately 1 km and a gradient of up to 26%. Participants will hike 1 of the 2 test routes shortly after the laboratory measurement. Based on these data and in combination with the capacity of maximal oxygen uptake (VO_2max_) derived in the laboratory setting, a statistical model for the prediction of VO_2max_ will be developed.

#### Secondary Outcome

During the 8-km hiking tour, the cardiovascular load will be measured on defined hiking sections (ascents, descent, and plain levels). Based on the individually predicted VO_2max_ on the 1-km CTTT, the estimated cardiovascular load (heart rate or oxygen cost) will be defined and visualized in a digital map using 3 different intensity levels (low, moderate, and high intensity).

#### Accuracy of Heart Rate Measurements

Each participant’s heart rate will be measured simultaneously from at least two different sources during every part of the study. We aim to evaluate the accuracy of the wrist-based Garmin Vivoactive 4 smartwatch data and Garmin chest strap data considering the gold standard 12-lead ECG data measured during the treadmill test. Further, the wrist-based measurement accuracy during the field tests will be compared with the chest strap measurement accuracy.

#### Comparison of Resting Evaluation Versus Exercise Test for Exercise Capacity

We will measure the maximal exercise capacity (VO_2max_) during the use of the modified Bruce protocol on the treadmill. As we will use the portable spirometry device K5 from Cosmed, we will be able to use the DMC method instead of the breath-by-breath measurement.

As we will obtain measured VO_2max_ values, we will be able to compare those results with estimations for this parameter, which we will obtain with the Polar Fitness Test. This test is a 5-min resting evaluation performed with a chest strap and the Polar beat app. Based on the measured heart rate variability and added data like age, gender, weight, height, and fitness classification, the app will estimate the individual’s VO_2max_.

In addition, we will obtain VO_2max_ estimation based on the outdoor hikes that involve recording with the Garmin Vivoactive 4 smartwatch.

We aim to evaluate the validity of the 2 VO_2max_ estimations.

#### Physical Activity and Quality of Life

We will use the IPAQ short form to assess participants’ physical activity levels. The questionnaire captures vigorous, moderate, and walking activities and sitting times of the previous 7 days.

The SF-36 will be used to assess health-related quality of life. It contains 36 items grouped into the following 8 dimensions: physical functioning, physical role, body pain, general health, vitality, social functioning, emotional function, and mental health.

### Statistical Analysis

#### Sample Size Calculation

The sample size calculation was done with G*Power Software (Version 3.1.9.7; Heinrich-Heine University) [18,19]. With α=.05, β=.1, and a middle effect size of *f²*=0.15, a strong model should be created. The calculation of maximal oxygen uptake could be performed using a linear multiple regression model with 6 predictors. For a model power of 0.90, 123 participants would be required to finish the study. To make sure we reach the number we chose, we added a 15% dropout rate and set the target sample size to 144 participants, with a balanced distribution in Austria and Germany.

#### Descriptive and Statistical Significance

Statistical evaluations of the hikes (per region or participant) will be performed. Since this study aims to develop a model for calculating exercise capacity during a hiking field test, the statistical analysis will be performed on a per protocol basis, thus including only participants who complete the lab test, the 1-km CTTT, and the 8-km hike. Statistical analyses will be performed using SPSS version 24.0 (SPSS Inc) and R version 4.1.0 (The R Project for Statistical Computing).

#### Visualization of Exercise Capacity on Hiking Trails

To visualize the analysis results, the collected data on the 8-km hiking trails in Austria and Germany will be used to determine the average exercise capacity for defined hiking trail sections. Therefore, accumulated heart rate and maximal oxygen uptake will be map matched on the hiking trail sections representing different intensities during the hike.

#### Algorithm Development

Algorithm development is based on the objectives of the analyses, which are as follows: (1) The implementation of an estimation model for exercise capacity; (2) The definition of the hiking trail sections; and (3) The recommendation of intensity ranges via visualization of the hiking trail sections of the 8-km hike based on the capacity estimation of the 1-km CTTT.

#### Estimation Model for Exercise Capacity

As the first step in model development, the data collected in the laboratory and the results from the 1-km CTTT and 8-km hike will be analyzed descriptively to identify anomalies in the data, such as outliers or data loss due to possible device crashes. Based on these analyses, the data for Austria and Germany will be cleaned.

Following the principle “from the lab to the field,” the first estimation model will be developed based on features collected in the laboratory to verify how accurate and reliable VO_2max_ can be estimated. When the proof of concept works on the laboratory data, an in-field estimation model will be created based on the CTTT measurements to see if VO_2max_ from the laboratory can be determined based on the data collected on the CTTT. The derived estimation models will be validated with cross-validation and evaluation metrics (eg, adjusted R-squared and mean absolute error).

#### Definition of Hiking Trail Sections

The definition of hiking trail sections will be a combination of expert-based labeling and a data-driven approach. Sports scientists will mark relevant waypoints due to their expected change in intensity. The data-driven approach will use data from some participants on the 8-km hikes to determine the remarkable difference in intensities. The final definition will combine both approaches and will determine the section points.

#### Visualization of Hiking Trail Sections With Recommended Intensity

For each 8-km hike, the recommendations will include the impersonalized visualization of expected intensities and the personalized recommended intensity. The collected study data will determine the expected intensities and will be georeferenced to the hiking trail sections. The recommended intensity ranges for each hiking trail section of the 8-km hikes will be determined based on the capacity estimation of the CTTT.

## Results

The project “Connect2Move” was funded in December 2019 by the European Regional Development Fund (INTERREG V-A Programme Austria-Bavaria – 2014-2020; Project Number AB296). “Connect2Move” started in January 2020 and runs until the end of June 2022. By the end of April 2022, 162 participants were tested in the laboratory, and of these, 144 were tested outdoors. The data analysis will be completed by the end of June 2022, and results are expected to be published by the end of 2022.

## Discussion

This paper describes a protocol created by a multidisciplinary team of physicians, sports scientists, data scientists, and tourist experts to develop a standardized CTTT, which can evaluate the hiking-specific exercise capacity and hence the fitness level of hikers. This project focuses on hiking, as it is one of the most common outdoor activities in the alpine region and can be performed throughout the year. This outdoor activity encourages people to be physically active while spending time in nature. Besides greater physical fitness, hikers may also experience benefits from spending time in a natural environment, such as decreased blood pressure and stress levels, enhanced immune system, restored mental and emotional well-being, and improved general well-being [20,21]. To further promote this form of outdoor activity and raise awareness of the prevention of the overestimation of one’s fitness level, this study aims to develop an easy-to-performance fitness test located directly in popular hiking areas. Why should a new physical fitness test be developed when the literature reports several test protocols for field tests to evaluate physical fitness or exercise capacity (VO_2max_)? Tests like the 6-min walking test [22,23] and the Urho Kaleva Kekkonen (UKK) walking test [24] are regularly used by numerous professionals (physicians, sports scientists, coaches, etc) and are well established in the clinical setting. However, these tests are used mainly for patients with underlying diseases like cardiovascular or pulmonary diseases. Existing walking tests are usually performed on flat surfaces (eg, athletic tracks) and not on an inclined slope, making it hard to give recommendations for hiking exercises, since hiking track profiles range from plain surfaces to inclining and declining surfaces. Furthermore, most popular hiking areas cannot provide long enough flat tracks to perform such tests. Therefore, this study aims to develop a hiking-specific CTTT, which will be validated with a standardized laboratory exercise test.

Similar to our study, Chiaranda et al [25] developed an equation for a 1-km submaximal treadmill test for patients with cardiovascular diseases. They recommend it as a possible substitute for maximal exercise testing at laboratories in rehabilitation and health and fitness facilities where expense, space, time, and personnel needed to carry out these cardiopulmonary tests are limited. They also tried to reproduce this 1-km flat submaximal walking test in an outdoor setting with similar results [26]. However, their equations only apply to cardiac patients. We set out to develop an equation for healthy individuals interested in hiking, which is one of the most common leisure activities in mountainous areas. We aim a step further as we hope to integrate this equation into existing hiking apps to deliver individual recommendations based on the performance at a standardized CTTT. In addition, the Connect2Move project hopes to disseminate the characteristics of the developed equation and CTTT throughout interested mountainous areas.

As the interest in green tourism and holidays focusing on health increases, we aim to deliver cost-effective easily accessible tools that facilitate individual recommendations for hiking tours and help avoid overexertion, resulting in fewer cardiovascular events while hiking.

## Appendix

Multimedia Appendix 1 External peer-review report from the ESI Funds 2014-2020, INTERREG V-A Austria-Germany/Bavaria 2014-2020 (Munich, Germany).

Multimedia Appendix 2 Machine translation (by Google) of the external peer-review report.

## Abbreviations

CTTT: cardio trekking test trail
DMC: dynamic mixing chamber
ECG: electrocardiography
IPAQ: International Physical Activity Questionnaire
RPE: rate of perceived exertion
SF-36: 36-Item Short Form Health Survey
