# Supplementary material for: Valorization of Natural Cardio Trekking Trails Through Open Innovation for the Promotion of Sustainable Cross-generational Health-Oriented Tourism in the Connect2Move Project: Protocol for a Cross-sectional Study
Source: JMIR Res Protoc. 2022 Jul 13;11(7):e39038. doi: 10.2196/39038 (PMC9330191; doi:10.2196/39038)
Supplement: Multimedia Appendix 1 [file resprot_v11i7e39038_app1.pdf]

Assessment Check

Project identification

Project ID		AB296	
Project title		Inwertsetzung von natürlichen Cardio-Trekking-Wanderwegen durch Open Innovation zur nachhaltigen Förderung des generationsübergreifenden, gesundheitsorientierten Tourismus	
Project acronym		Connect2Move	
Name of the lead partner organisation/English		Technische Universität München	
Project duration	Start date	2020-01-01	Nr. of months
	End date	2022-06-30	30 months
Programme priority		2 Erhaltung und Schutz der Umwelt sowie Förderung der Ressourceneffizienz	
Programme priority specific objective		3 Inwertsetzung des Natur- und Kulturerbes im Hinblick auf eine nachhaltige, grenzüberschreitende touristische Entwicklung	

Project summary

Please give a short description of the project:
Issue:

- Which issue/challenge will the project address?
- Where does it currently occur in the NWE Programme area?
- Where will the project address it (territory)?

Change:

- How much will the project change the current situation (please quantify the objective in volume or value)?

Novelty:

- What is new/original about the approach taken to achieve this change?

Outputs:

- Which main outputs/pilots/investments will the project produce to achieve this change?

Long term effects:

- How and where does the project plan to sustain and further roll-out its main outputs/pilots/investments after the end of the project?

DE	**Zielsetzung:** Inwertsetzung von natürlichen und evidenzbasierten Cardio-Trekking-Wegen durch Open Innovation Methoden zur nachhaltigen Förderung eines generationsübergreifenden, gesundheitsorientierten Tourismus. **Inhalte:** U nter besonderer Berücksichtigung der Alpenregion, inklusive ihres kulturellen Erbes (z. B. Ökomodellregion (D), Bergsteigerdörfer (D), Römerregion (D), Almen- und Höhenwege (AT)) sollen bestehende Wanderwege zu Themenwegen gestaltet und digital neu kartographiert werden, in der neben der gewohnten Beschreibung der Länge, Höhenmeter, Wegbeschaffenheit und Dauer auch die kardiovaskulären Belastungsintensitäten gekennzeichnet werden. Ein Parameter für die Belastungsintensität ist die Herzfrequenz. Die Kennzeichnung der Naturwege und die Einordnung dieser erfolgt digital gestützt (als Ampelsystem, von rot "intensiv" zu grün "leichte Intensität") in bestehenden Wander-/TourismusApps. Zur Inwertsetzung und Implementierung in den teilnehmenden Gemeinden wird ein Open Innovation Ansatz gewählt, der sportwissenschaftlich und medizinisch begleitet wird und die regionalen Stakeholder, als auch die Bevölkerung einbindet. Es werden zwei grenzübergreifende, klimafreundliche und naturnahe Konzepte zur Bewegungsförderung/Cardiotrekking in den Alpen entwickelt. Die Konzepte dienen der Steigerung des ganzjährigen, sanften Gesundheitstourismus und auch der Förderung der individuellen Gesundheitskompetenz für Touristen u. Einheimische. **Ausblick:** *Connect2Move* fördert neue Tourismusinnovationen und -angebote mit "Herzgesundheit", durch die sanfte Inwertsetzung von Wegen mit kulturellem Erbe zu Cardiotrekking-Wegen zur Bewegungsförderung und gleichzeitigen Wissensvermittlung. *Connect2Move* fördert langfristig einen klimaneutralen Tourismus, steigert die Besucherzahlen in der Nebensaison, durch ein nachhaltiges, naturnahes "Herzgesundheitskonzept" (z. B. Zertifizierung von Gemeinden, Hotels).

Checklists

Checklist 13: GS - Zusammenfassende Prüfung

Question	Question Type	Answer	Comment	User	Confirmed
Durchschnittliche Punktezahl in den RK-Bewertungen	number	19,00	Fachlich-inhaltliche Qualität: hoch- sehr hoch Grenzüberschreitende Ausarbeitung: gut-sehr gut geeignet	312	Confirmed
Punktezahl in der GS-Bewertung	number	13,00	Hoher Beitrag zum SZ Indirekt Mäßiger Beitrag zum EI Mäßiger Beitrag zum OI	312	Confirmed

Die Bewertungen der Regionalen Koordinierungsstellen sind plausibel und nachvollziehbar.	yesno	Yes	Die Bewertungen der Regionalen Koordinierungsstellen sind nachvollziehbar und plausibel.	312	Confirmed
Bemerkungen zum Ergebnis der Antragsprüfung	input		Abgleich Rechtsstatus: LP; PP2; PP5; PP6: Angaben gemäß Antrag ident mit RK-Bewertung PP3: öffentlich finanziert, Gesellschaftsform privat – Anpassung im eMS erforderlich (Google Recherche erforderlich –wird durchgeführt und dokumentiert (eMS)) PP4: Angaben gemäß Antrag nicht ident mit RK Bewertung; gemäß RK –Bewertung ist PP4 eine öffentliche Organisation (Google Recherche erforderlich –wird durchgeführt und dokumentiert (eMS)) Beihilfenrechtliche Relevanz: LP; PP2; PP3; PP4; PP5; PP6: keine Beihilfenrechtliche Relevanz gemäß RK-Bewertung Ausrüstungskosten: LP, PP3: Ausrüstungskosten liegen vor, sind zur Gänze ff. PP2: Server Hosting Gebühren evtl. Ausrüstungskosten (finale Abklärung bis zum BA) PP4: geringwertige Wirtschaftsgüter zur Gänze ff Bei allfälliger Genehmigung sind Korrekturen im Antrag gemäß GS und RK Bewertung vorzunehmen. Überlegungen zur Entscheidung: Im Rahmen des Projektes wird das Natur- und Kulturerbe im Grenzraum SBG-OBB schonend inwertgesetzt, durch die Konzeptentwicklung für einen sanften, evidenzbasierten, naturnahen Gesundheitstourismus. Die grenzüberschreitende Ausarbeitung wird als sinnvoll erachtet aufgrund gleicher Herausforderungen im Alpenraum. In den Bewertungen durch die Regionalen Koordinierungsstellen wird vor allem die Betrachtung eines grenzüberschreitenden Raumes einer grenzübergreifenden Destination per se als sehr wertvoll gesehen. Der nachhaltige, gesundheitsorientierte	312	Confirmed

			Tourismus als grenzüberschreitendes, verbindendes Thema stärkt die Region. In einer fachlichen Stellungnahme wird auch betont, dass durch das Projekt ein deutlicher Mehrwert für den Gesundheitstourismus, der immer mehr an Bedeutung gewinnt, generiert wird.		
