# Supplementary material for: Valorization of Natural Cardio Trekking Trails Through Open Innovation for the Promotion of Sustainable Cross-generational Health-Oriented Tourism in the Connect2Move Project: Protocol for a Cross-sectional Study
Source: JMIR Res Protoc. 2022 Jul 13;11(7):e39038. doi: 10.2196/39038 (PMC9330191; doi:10.2196/39038)
Supplement: Multimedia Appendix 2 [file resprot_v11i7e39038_app2.pdf]

Machine Translated by Google

Assessment Check

Project identification

Project ID		AB296	
Project title		Valorization of natural cardio trekking trails through open innovation for the sustainable promotion of intergenerational, health-oriented tourism	
Project acronym		Connect2Move	
Name of the lead partner organisation/English		Technical University of Munich	
Project duration	Start date	2020-01-01	Nr. of months
	End date	2022-06-30	30 months
Programme priority		2 Preservation and protection of the environment and promotion of resource efficiency	
Programme priority specific objective		3 valorization of natural and cultural heritage with a view to a sustainable, cross-border tourism development	

Machine Translated by Google

Project summary

Please give a short description of the project:<br>Issue:<br>• Which issue/challenge will the project address?<br>• Where does it currently occur in the NWE Programme area?<br>• Where will the project address it (territory)?<br>Change:<br>• How much will the project change the current situation (please quantify the objective in volume or value)?<br>Novelty:<br>• What is new/original about the approach taken to achieve this change?<br>Outputs:<br>• Which main outputs/pilots/investments will the project produce to achieve this change?<br>Long term effects:<br>• How and where does the project plan to sustain and further roll-out its main outputs/pilots/investments after the end of the project?	
OF	**Objective:** Valorization of natural and evidence-based cardio trekking paths through open innovation Methods for the sustainable promotion of cross-generational, health-oriented tourism. **Contents:** U With special consideration of the Alpine region, including its cultural heritage (e.g. eco-model region (D), Mountaineering villages (D), Roman region (D), Almen and Höhenwege (AT)) existing hiking trails are to be converted into themed trails be designed and digitally re-charted, in which, in addition to the usual description of the length, altitude, The condition and duration of the path and the cardiovascular stress intensities are also marked. A parameter for exercise intensity is heart rate. The nature trails are marked and classified digitally supported (as a traffic light system, from red "intensive" to green "light intensity") in existing hiking/tourism apps. An open innovation approach is chosen for valorization and implementation in the participating communities, which is accompanied by sports science and medicine and the regional stakeholders, as well as the population involves. Two cross-border, climate-friendly and natural concepts for Movement promotion/cardio trekking developed in the Alps. The concepts serve to increase the year-round, gentle health tourism and also the promotion of individual health competence for tourists and the like. Local people. **Outlook:** *Connect2Move* promotes new tourism innovations and offers with "heart health", through the gentle valorization of paths with cultural heritage to cardiotrekking paths to promote physical activity and simultaneous knowledge transfer. *Connect2Move* promotes long-term climate-neutral tourism, increases Visitor numbers in the off-season, through a sustainable, natural "heart health concept" (e.g. certification from municipalities, hotels).

Checklists

Checklist 13: GS - summary test

Question	Question Type	Answer	Comment	User Confirmed
average score in the RK ratings	number	19,00	Technical content quality: high- very high border crossing Elaboration: good-very good suitable	312 Confirmed
score in the GS rating	number	13,00	High contribution to the SZ Indirect Moderate contribution to EI Moderate contribution to HI	312 Confirmed

The assessments of the regional coordination offices are plausible and understandable.	yesno	Yes	The reviews of regional Coordination points are comprehensible and plausible.	312 Confirmed	
Comments on the result of the application review	input		Comparison of legal status: LP; PP2; PP5; PP6: Information according to the application is identical to the RK assessment PP3: publicly financed, private company form - adjustment in the eMS required (Google research required - will be carried out and documented (eMS)) PP4: Information according to the application is not identical to the RK assessment; According to the RK assessment, PP4 is a public organization (Google research required - will be carried out and documented (eMS))<br><br>Relevance under state aid law: LP; PP2; PP3; PP4; PP5; PP6: No relevance to state aid according to RK assessment Equipment costs: LP, PP3: Equipment costs are available, are fully ff. PP2: Server hosting fees, possible equipment costs (final clarification up to BA) PP4: Low-value assets are fully ff Make corrections in the application according to GS and RK assessment. Considerations for the decision: As part of the project, the natural and cultural heritage in the SBG-OBB border area is carefully valorised, through the development of a concept for gentle, evidence-based, natural health tourism. the<br><br>Cross-border elaboration is considered useful due to the same challenges in the Alpine region. In the evaluations by the regional coordination offices, the consideration of a cross-border area of a cross-border destination is seen as very valuable per se. The sustainable, health-oriented<br><br>tourism as	312 Confirmed	

			Tourism strengthens as a cross-border, connecting topic the region. A technical statement also emphasizes that the project generates significant added value for health tourism, which is becoming increasingly important.		
